# Longitudinal analysis of ultrasonic vocalizations in mice from infancy to adolescence: Insights into the vocal repertoire of three wild-type strains in two different social contexts

**DOI:** 10.1371/journal.pone.0220238

**Published:** 2019-07-31

**Authors:** Tatiana Peleh, Ahmed Eltokhi, Claudia Pitzer

**Affiliations:** 1 Interdisciplinary Neurobehavioral Core, Heidelberg University, Heidelberg, Germany; 2 Research Group of the Max Planck Institute for Medical Research at the Institute of Anatomy and Cell Biology, Heidelberg University, Heidelberg, Germany; University of Missouri Columbia, UNITED STATES

## Abstract

Ultrasonic vocalizations (USV) are emitted by mice under certain developmental, social and behavioral conditions. The analysis of USV can be used as a reliable measure of the general affective state, for testing the efficacy of pharmacological compounds and for investigating communication in mutant mice with predicted social or communication deficits. Social and communication studies in mice have focused mainly on the investigation of USV emitted by neonatal pups after separation from the dam and during social interaction between adult males and females. Longitudinal USV analysis among the different developmental states remained uninvestigated. In our study, we first recorded USV from three inbred mouse strains C57BL/6N, DBA/2 and FVB/N during the neonatal stages after separation from the littermates and then during a reunion with one littermate. Our results revealed significant strain-specific differences in the numbers and categories of USV calls. In addition, the USV profiles seemed to be sensitive to small developmental progress during infancy. By following these mice to the adolescent stage and measuring USV in the three-chamber social test, we found that USV profiles still showed significant differences between these strains in the different trials of the test. To study the effects of social context on USV characteristics, we measured USV emitted by another cohort of adolescent mice during the direct social interaction test. To this end, this study provides a strategy for evaluating novel mouse mutants in behavioral questions relevant to disorders with deficits in communication and sociability and emphasizes the important contribution of genetics and experimental contexts on the behavioral outcome.

## Introduction

Wild-type mice emit ultrasonic vocalizations (USV) with frequencies higher than 20 kHz, which cannot be detected by human ears. USV are produced in different situations including aversive affective states and negative situations [1–3], social interaction to enhance social bonding in juvenile [4] and adult mice [3, 5–7] and appetitive situations such as rough-and-tumble play in juvenile and courtship in adult mice [8, 9]. Moreover, pups separated from dam and littermates during their first two weeks of life emit a large number of USV to trigger the mother’ retrieval behavior [10–12] and in response to body temperature dropping, handling and specific smells. In particular, recording USV in mice has great potential to provide a model system for the investigation of communication in wild-type and mutant mice with neuropsychiatric-like phenotypes [13, 14]. Alterations of USV have been reported in several mouse models of neuropsychiatric disorders including autism [15–24] and schizophrenia [13, 25]. These studies have proved that studying USV can help to understand the mechanisms of human communication and associated disorders. Moreover, recording USV is a useful tool for testing the efficacy of pharmacological compounds [26–28] and for monitoring the general health of mice [5].

USV can be classified into ten different patterns with distinctive temporal-structural characteristics designated as frequency steps, harmonics, composite, short, complex, chevron, flat, downward, upward, two-syllable [4, 6, 29]. The specific sequential pattern of calls is suggested to convey specific information; e.g., female mice preferentially approach specific patterns of USV emitted by males [30]. However, other important factors including number, rate, duration and peak frequency of USV are still important cues used by mice to interpret the nature of the social interaction [31] and these factors are dependent on age and genetic background [32–35]. The detailed characterization of USV properties requires many steps including recording, call detection, call type classification and clustering [36].

In the present study, we compared three inbred mouse strains, C57BL/6N, DBA/2 and FVB/N, in respect to the basic USV parameters including the number of calls, frequency, duration and amplitude as well as the call structure based on a previously published USV category scheme [6]. We have selected these strains since they are typically used as background strains for breeding genetically modified mice and are able to show robust social interaction ability [37]. Although they represent the most used standard controls for many behavioral studies, each strain can display different characteristics under certain behavior paradigms [38–42]. For example, DBA/2 mice perform poorly in several hippocampus-dependent contextual and spatial learning tasks compared to C57BL/6 mice [43]. Moreover, DBA/2 mice have a decreased locomotor activity in the open field task compared to FVB/N mice [40]. On one hand, the FVB/N strain is preferable for transgenic analysis because its fertilized eggs contain large and prominent pronuclei, which facilitates the microinjection of DNA [44]. On the other hand, this strain shows high anxiety, aggressive behavior and disturbed circadian rhythm [45]. To this end, we first assessed possible differences in the USV properties between neonatal pups of the selected mouse strains after their separation from the dam and littermates. Moreover, we checked the effects of small developmental progress during infancy as well as the reunion with a littermate on the patterns of the USV emissions. Furthermore, we measured USV from the same mice at the adolescent stage in the three-chamber social test [46]. Additionally, we tested the effects of social context on the USV patterns by measuring USV of the three selected mouse strains in the direct social interaction test.

This study offers a deeper insight into the discrete functions, individual calls and calls sequences within the diversity of the vocal repertoire, which can be used for further understanding of the acoustic signals associated with social interactions in mice and in testing the role of motivational states in the emission of USV. Moreover, it can be used as a reference for testing mice with predicted communication deficits or for testing the efficacy of drugs treating social deficits by considering the crucial importance of the developmental state, social context and genetic background in the regulation of different behaviors.

## Materials and methods

### Animals and housing conditions

**Fig 1:**
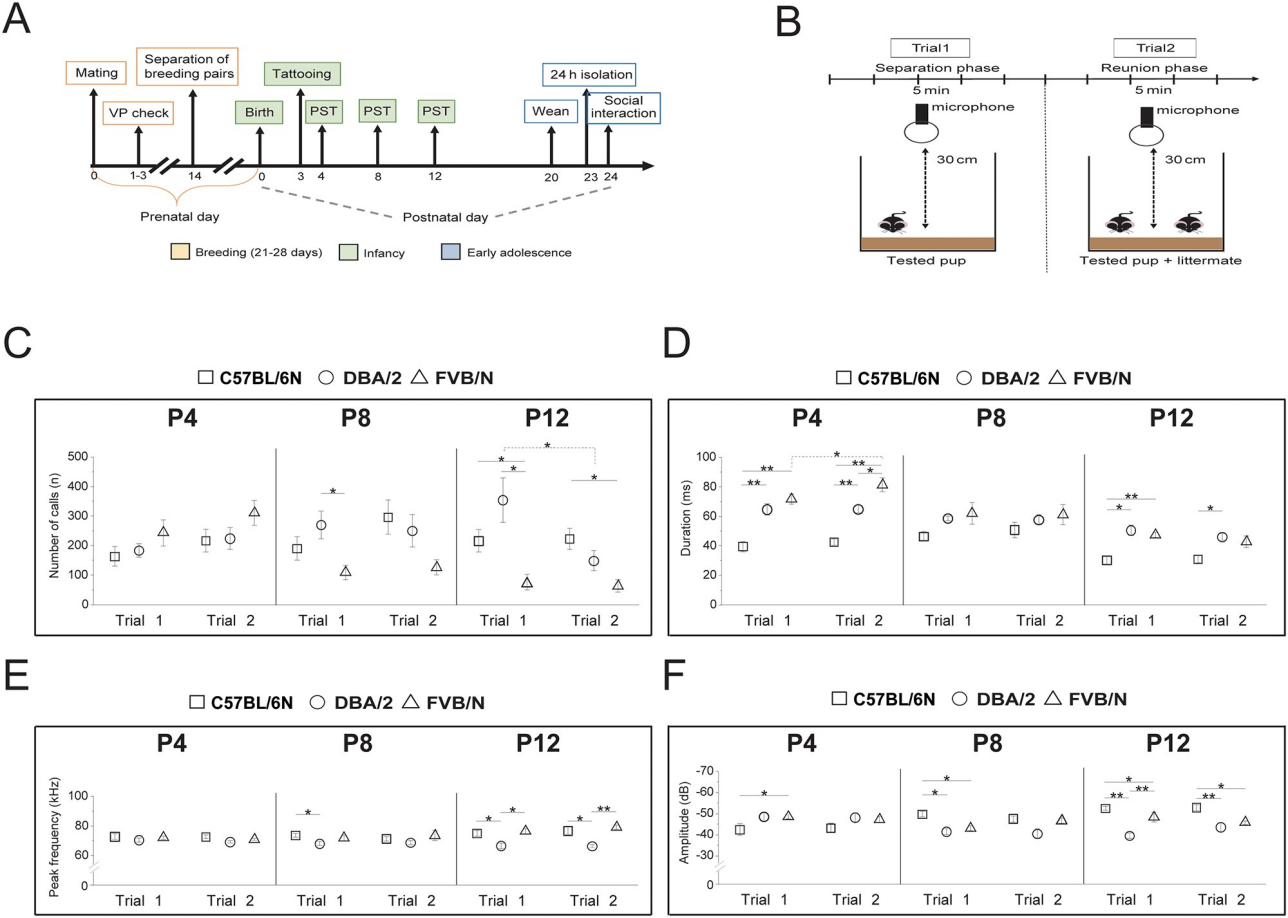
USV analysis of C57BL/6N, DBA/2 and FVB/N pups during infancy after separation and reunion with a littermate. **(A)** A schematic drawing of the experimental protocol. The recording of USV during infancy was done at P4, P8 and P12. The recording of USV for adolescent mice was done at P24 after 24 h of isolation of mouse from the littermates, VP: vaginal plug, PST: pup separation test. **(B)** A schematic drawing of the experimental setup for USV recording during infancy. USV were recorded for pups during separation phase (trial 1) for 5 min followed by a reunion with a littermate (trial 2). **(C-F)** USV analysis including numbers **(C)**, durations **(D)**, frequencies **(E)** and amplitudes **(F)** of calls at P4, P8 and P12 during separation phase (trial 1) and reunion with a littermate (trial 2); n = 7 for C57BL/6N (2 males and 5 females), n = 8 for DBA/2 (4 males and 4 females) and n = 11 for FVB/N (6 males and 5 females), all data as mean ± SEM (two-way ANOVA, *p< 0.05, **p< 0.01).

C57BL/6N, DBA/2 and FVB/N female and male mice were purchased from Janvier Laboratories (Le Genest- Saint- Isle, France) at the age of 8 weeks. After ten days, female and male mice were paired. Then, females were isolated from males after ten days of pairing, housed individually and inspected daily for pregnancy and delivery. The day of birth was considered as postnatal day (P) 0. Pups were marked by paw tattoo at P3 using a non-toxic animal tattoo ink (Ketchum permanent tattoo green paste, Ketchum Manufacturing, Inc., Brockville ON Canada). The ink was injected subcutaneously through a 25 gauge hypodermic needle tip into a toe of a paw. At P20, mice were weaned, and the littermates from the same sex were grouped in the same cage (three to four per cage) (Fig 1A). All animals had *ad libitum* access to food and water under a standard 12 h light/dark cycle (7:00 pm—7:00 am) with a regulated ambient temperature of 22 °C +/- 1 and at a relative humidity of 40–50%. All experiments were conducted in strict compliance with the National Institutes of Health Guidelines for the Care and Use of Laboratory Animals and approved by the Regional Council in Karlsruhe (Regierungspräsidium Karlsruhe, Germany; Animal Ethic Protocol 35–9185.81/G-129/15).

### USV analysis during infancy after separation and reunion with a littermate

USV were recorded in infant mice at P4, P8 and P12. At each developmental stage, the testing pup was separated from the littermates and placed in an empty glass container (6 x 9.5 x 7.5 cm) with the floor covered with bedding material. The container was placed inside a box with the walls covered with Styrofoam to attenuate surrounding noise. USV were recorded for five min (separation trial 1) which was immediately followed by placing one littermate next to the testing pup for an additional five min (reunion trial 2) (Fig 1B). Before placing the pup in the glass container, it was weighed, and the body temperature was measured by gently applying a thermal probe on the belly (digital thermometer Bosotherm medical, Boso, Jungingen, Germany). After the USV measurement, the pup was immediately returned to its home cage. The same paired pups were used among the 3 days of recordings. The numbers of pups used in this study were 7 for C57BL/6N (2 males and 5 females), 8 for DBA/2 (4 males and 4 females) and 11 for FVB/N (6 males and 5 females).

### USV analysis in adolescent mice during the three-chamber social test

USV were recorded among adolescent mice between P24 and P29. The pairs of tested mice were from the same sex and litter. In our adapted protocol, the three-chamber social test was designed to assess the social ability with a familiar conspecific and with a stranger adult mouse. USV were measured from all chambers in parallel using one microphone placed 30 cm above the arena floor of the middle chamber. The three-chamber social apparatus was made up of a transparent plexiglas box (20 x 61 x 40 cm) with three compartments including sliding doors (5 x 8 cm) between the compartments. The top of each compartment was closed with a plexiglas top cover. A wire mesh cylinder was placed in the left and right compartments. The test was subdivided into three trials, 10 minutes each, with a 15 min intertrial interval. In trial 1 (habituation), the testing mouse, isolated from the home cage for 24 h, was introduced into the apparatus with free access to all compartments (Fig 3A). In trial 2 (social recognition), a littermate mouse was placed into the right wire mesh cylinder and the subject mouse was allowed to explore all compartments. In trial 3 (social discrimination), an unfamiliar adult mouse from a different strain was placed into the left mesh wire cylinder: one DBA/2 mouse for C57BL/6N strain; one FVB/N for the DBA/2 strain and one C57BL/6N mouse for the FVB/N strain, and the subject mouse was again allowed to explore freely all compartments. The numbers of adolescent mice used were 7 for C57BL/6N (2 males and 5 females), 8 for DBA/2 (4 males and 4 females) and 11 for FVB/N (6 males and 5 females).

### USV analysis in adolescent mice during the direct social interaction test

USV were recorded among adolescent mice between P24 and P29. The pairs of tested mice were from the same sex and the same litter. One adolescent mouse was isolated from the home cage for 24 h in a separate colony housing room. The social test included 2 trials with trial 1 serving as a habituation phase by placing one mouse from the colony into a white acryl open-field box (40 × 40 × 40 cm) for 2 min. In trial 2, the sibling mouse was placed gently next to the isolated mouse for 5 min (social interaction trial). USV were recorded along both trials using a microphone placed 30 cm above the arena floor (Fig 4A). The numbers of tested adolescent mice were 25 for C57BL/6N (14 males and 11 females), 14 for DBA/2 (7 males and 7 females) and 18 for FVB/N (7 males and 11 females).

### The USV recording and analysis

The USV recording and analysis were conducted with an equipment and a software from Avisoft Bioacoustics (Berlin, Germany). The acoustic signals were recorded, amplified and digitized at 250 kHz with a 16-bit resolution by the Ultrasound Gate 416 Hb USB audio device and ultrasonic condenser microphones CM16/CMPQ were placed 30 cm above the floor of the testing area. For acoustical analysis, signals saved as wave-files were transferred to the Avisoft SLabPro version 5.2.07 (Avisoft Bioacoustics, Berlin, Germany), and a Fast Fourier Transformation was conducted (512 FFT-length, 100% frame Hamming window and 75% time window overlap). Spectrograms displaying frequency versus time were produced with a 488 Hz frequency resolution and 0.512 ms time resolution. The number of calls and a wide range of acoustic parameters were automatically measured using the automatic whistle tracking algorithm with a minimum duration of 3 ms and a hold time of 10 ms. A cut-off filter of 15 kHz and a post filter (minimum duration of 1 ms and entropy of 0.5) were applied. Accuracy of the number of calls was verified manually and existing extraneous noise was removed from the sonograms. USV categories were defined manually according to a previously published classification scheme [6].

### Statistical analysis

Prism 6 software (GraphPad Software) and Microsoft Office Excel software were used for data analysis. For all measurements, data were calculated and presented as mean ± SEM. The two-way ANOVA test with strain and sex as the two factors was performed to assess statistical significance. According to Bonferroni correction for multiple testing, *P*-values were corrected by multiplying the original p-values by the number of hypotheses (6 hypotheses for pups and adolescent mice in the direct social interaction test; 9 hypotheses for adolescent mice in the three-chamber social test). Corrected *P*-values ≤ 0.05 were considered significant.

## Results

### Body weight and temperature during infancy

In order to monitor the developmental progress in infant mice, the body weight was measured at postnatal day (P) 4, 8 and 12. All strains gained weight during the first 12 days after birth. However, DBA/2 pups were significantly smaller compared to C57BL/6N and FVB/N pups at the three investigated developmental stages (S1A Fig). Infant mice are unable to control their body temperature during the first days of life and therefore tend to suffer from hypothermia which is a major cause of infant death in nature. In order to control body temperature and ensure the standardization of the testing procedures, the body temperature was measured pre- and post-experimentally at all measurement days. At P4, C57BL/6N pups lost on average 3.46 ± 0.64 °C, DBA/2 pups 1.75 ± 2.04 °C and FVB/N pups 2.89 ± 0.55 °C after testing (S1B Fig). The temperature loss diminished for C57BL/6N mice at P8 indicating a fast improvement in the thermoregulation of the developing pups. However, for DBA/2 and FVB/N, the temperature loss diminished later at P12 (S1B Fig).

### Ultrasonic vocalization in pups

#### Effect of separation (trial 1) on the number of calls

Separated pups tend to emit USV which are suggestive for mother seeking behavior. At P4, there was no difference in the number of calls between C57BL/6N, DBA/2 and FVB/N pups (Fig 1C). At P8, the number of calls emitted by FVB/N pups was significantly lower than DBA/2 (p = 0.034) and lower than both C57BL/6N (p = 0.032) and DBA/2 (p = 0.015) at P12 (Fig 1C).

By comparing the three developmental states during infancy, we found that FVB/N pups emitted more calls at P4 than at P8 and P12 (p = 0.0084 and 0.0074, respectively) (S2 Fig). In contrast, C57BL/6N and DBA/2 pups did not show any difference in the number of calls between P4, P8 and P12 (S2 Fig).

#### Effect of reunion with a littermate (trial 2) on the number of calls

After a reunion with a littermate, the number of USV calls emitted by C57BL/6N pups slightly increased at P4 and P8. In contrast, the number of calls did not change at these stages for the DBA/2 strain. For the FVB/N strain, there was a slight increase in the number of calls at P4, but not at P8 or P12. Reunion with a second pup did not lead to a diminution of calls in all strains except for DBA/2 at P12 (p = 0.024) (Fig 1C).

#### Effect of separation and reunion on the call characteristics

Additionally to the number of calls, the acoustic characteristics like duration, frequency as well as the amplitude of the emitted USV were analyzed. The duration of calls ranged from 30 to 80 ms and varied across strains and developmental states in the separation trial 1 (Fig 1D). In all strains, the duration of calls declined significantly during infant development from P4 to P12 in both the separation and reunion trials (S2 Fig). The C57BL/6N pups showed the lowest duration across all developmental states in both trials (P4: trial 1: p = 0.008 vs DBA/2 and 0.007 vs FVB/N; trial 2: p = 0.0094 vs DBA/2 and 0.0068 vs FVB/N) (P8: trial: 1 p = 0.08 vs DBA/2 and 0.1 vs FVB/N; trial 2: p = 0.12 vs DBA/2 and 0.14 vs FVB/N) (P12: trial 1: p = 0.02 vs DBA/2 and 0.009 vs FVB/N; trial 2: p = 0.037 vs DBA/2 and 0.054 vs FVB/N), while the FVB/N pups showed the longest duration at P4 during both the separation and reunion trials (Fig 1D). By comparing trial 2 to trial 1, no gross changes in calls durations were present in any of the three tested strains (Fig 1D).

The frequency of USV calls ranged from 65 to 80 kHz throughout all tested postnatal stages and was not affected by the presence or absence of a littermate in all strains (Fig 1E). The DBA/2 pups emitted calls at a lower frequency than that emitted from the C57BL/6N and FVB/N pups with significance observed at P12 in both trials (Trial 1: p = 0.016 vs C57BL/6N and 0.017 vs FVB/N; trial 2: p = 0.013 vs C57BL/6N and 0.009 vs FVB/N) (Fig 1E). FVB/N pups showed an increasing call frequency between P4 and P12 in trial 2 (S2 Fig).

Measuring the amplitude of USV calls revealed that C57BL/6N pups emitted softer calls than DBA/2 and FVB/N pups at P12 in both trials (Trial 1: p = 0.005 vs DBA/2 and 0.02 vs FVB/N; trial 2: p = 0.007 vs DBA/2 and 0.014 vs FVB/N) (Fig 1F). These pups also displayed a significant upward trend of amplitude across the developmental states with calls becoming softer with age (S2 Fig). In contrast, the USV calls emitted by DBA/2 pups at P4 were softer than at P8 and P12 (S2 Fig). For all strains, the amplitude of USV did not change when a second littermate joined the first pup (Fig 1F).

#### Call categories in pups

**Fig 2:**
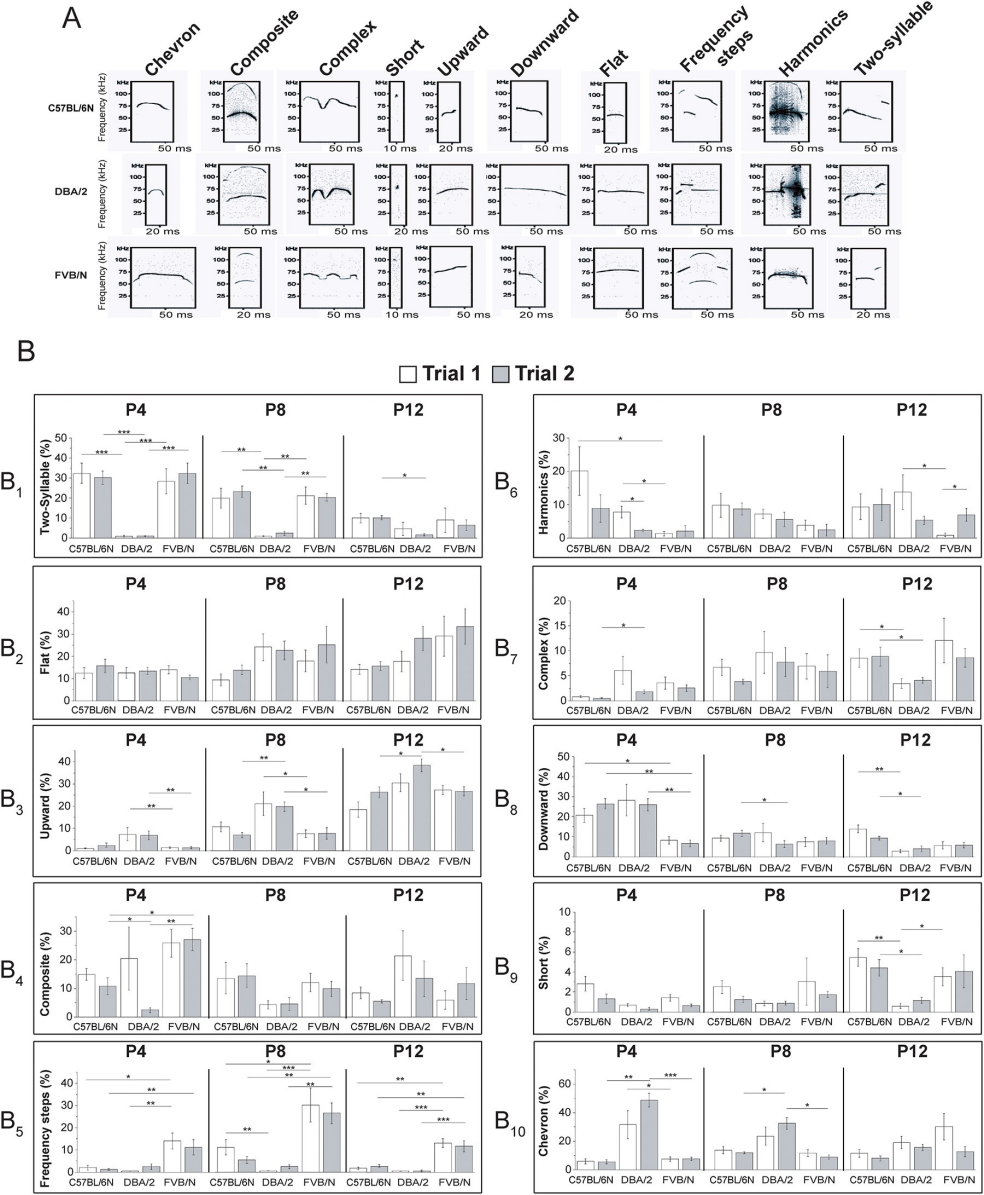
Analysis of USV categories of C57BL/6N, DBA/2 and FVB/N pups. **(A)** Representative sonograms of different calls categories with time in milliseconds (ms) represented on the X-axis and frequency in kHz on the Y-axis. **(B**_**1-10**_) Percentages of different call categories emitted by each strain at P4, P8 and P12 during separation (trial 1) and reunion with a littermate (trial 2); n = 7 for C57BL/6N (2 males and 5 females), n = 8 for DBA/2 (4 males and 4 females) and n = 11 for FVB/N (6 males and 5 females), all data as mean ± SEM (two-way ANOVA, *p< 0.05, **p< 0.01, ***p< 0.001).

According to their spectrographic shape, frequency and duration, USV were classified into ten categories [6]. These ten categories have been detected in the three strains and are presented with their relative frequencies and durations in Fig 2A. Our dissection of USV showed that the call categories were unique for each strain at specific developmental stages. The pattern emitted the most by C57BL/6N pups at P4 and P8 was the two-syllable pattern (25–30% of all patterns) (Fig 2B_1_). At P12, C57BL/6N mice emitted mostly the upward pattern (≃ 25%) (Fig 2B_3_). For DBA/2 pups, most of the calls produced at P4 and P8 were chevron (30–50%) (Fig 2B_10_), but upward at P12 (≃ 35%) (Fig 2B_3_). The most emitted patterns by FVB/N pups were two-syllable at P4 (≃ 30%) (Fig 2B_1_), frequency steps at P8 (≃25%) (Fig 2B_5_) and flat at P12 (≃ 30%) (Fig 2B_2_). The comparison of the individual call categories used by separated (trial 1) and reunited (trial 2) pubs did not reveal a socially-induced alteration of the call categories with the exception of a significant decrease in harmonic calls by DBA/2 P4 pups in trial 2 and its increase by FVB/N pups at P12 (Fig 2B_6_).

### USV in adolescent mice in the three-chamber social test

**Fig 3:**
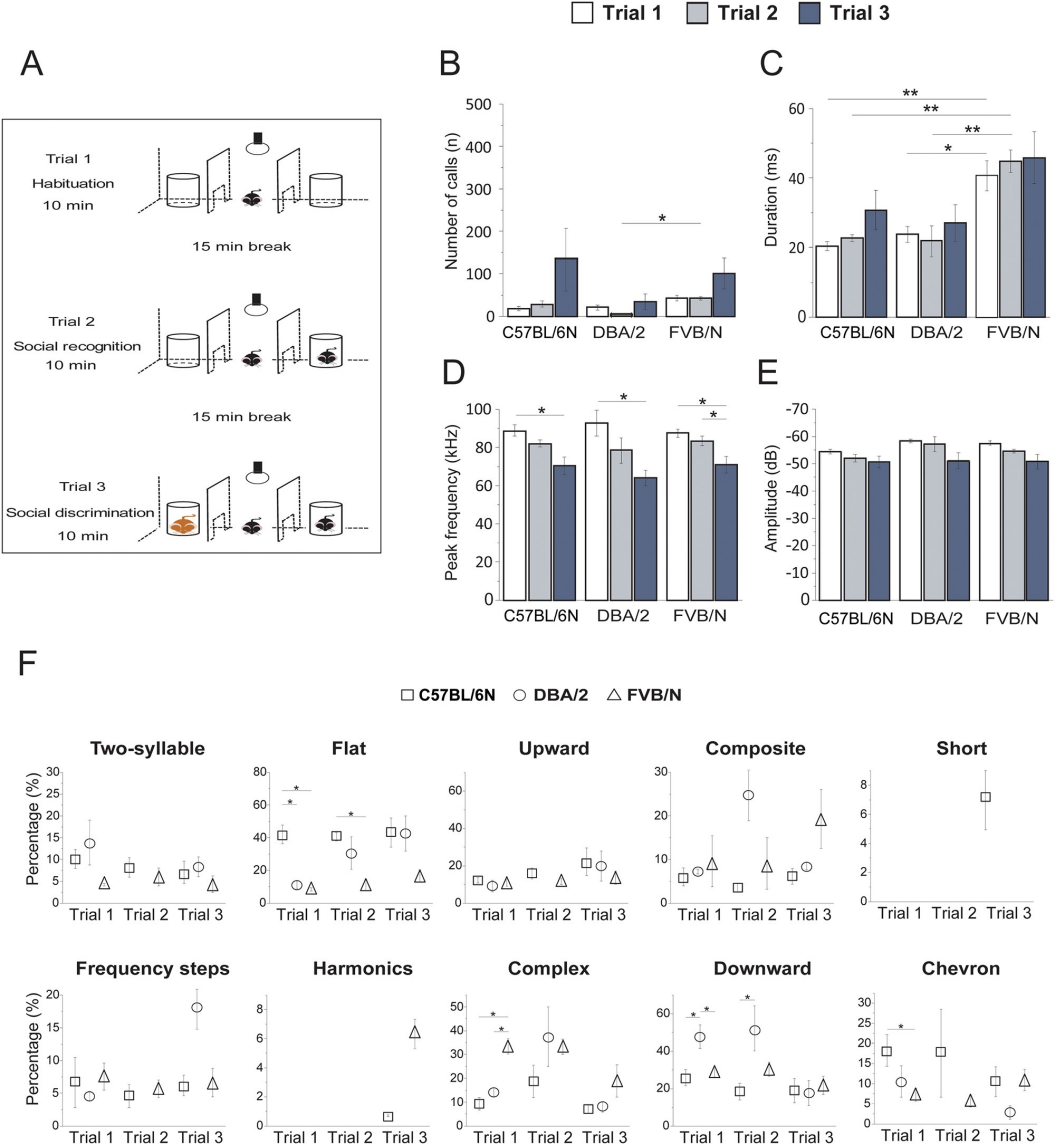
USV analysis of adolescent mice from C57BL/6N, DBA/2 and FVB/N strains during the three-chamber social test. **(A)** A schematic drawing of the experimental setup for USV recording in adolescent mice. USV were recorded for 10 min in the apparatus as a habituation phase (trial 1), followed by a social contact phase for 10 min with a same-sex littermate on the right compartment (trial 2) followed by another trial for 10 min with an adult unfamiliar mouse from another strain (trial 3). The intertrial interval between trials was 15 min. **(B-E)** USV analysis including number **(B)**, duration **(C)**, frequency **(D)** and amplitude **(E)** of calls during the three trials. **(F)** Percentages of different call categories emitted by each strain during each trial; n = 7 for C57BL/6N (2 males and 5 females), n = 8 for DBA/2 (4 males and 4 females) and n = 11 for FVB/N (6 males and 5 females), all data as mean ± SEM (two-way ANOVA, *p< 0.05, **p< 0.01).

The USV profiles of adolescent mice were also investigated using the three-chamber social interaction test (Fig 3A). Analyzing the number of calls within the 10 min habituation phase (trial 1) of the three chamber box revealed no differences between the three strains (Fig 3B). By presenting a familiar littermate in the right compartment (social recognition trial 2), the number of calls did not change significantly. However, the additional presence of an adult unfamiliar mouse (social discrimination trial 3) in the left compartment elicited an increase in the number of calls for all strains without reaching significance (Fig 3B). In all trials, FVB/N mice showed the longest calls durations with significant results in trial 1 (p = 0.008 vs C57BL/6N and 0.014 vs DBA/2 and trial 2 (p = 0.0074 vs C57BL/6N and 0.009 vs DBA/2) (Fig 3C). Interestingly, the frequency of the USV calls was significantly lower in all strains in the presence of the adult stranger mouse in trial 3 (C57BL/6N: p = 0.031 vs trial 1 and 0.054 vs trial 2; DBA/2: p = 0.037 vs trial 1 and 0.22 vs trial 2; FVB/N: p = 0.02 vs trial 1 and 0.034 vs trial 2) (Fig 3D). The amplitude of USV calls varied across trials and strains and tended to decrease with the introduction of the stranger mouse (Fig 3E). When considering the gender, no differences in the number, duration, frequency or amplitude of the calls were detected between male and female adolescent mice from all tested strains during all trials (S3 Fig).

The comparison between the USV calls emitted by adolescent mice in trials 1 and 2 of the three-chamber social test with the calls emitted in trials 1 and 2 during infancy revealed a significant decrease in the number of calls and their average durations in all strains during adolescence (Trials 1 and 2 of the three-chamber social test during adolescence vs trials 1 and 2 at P4: p<0.001 for all tested strains for both number and duration of calls). In contrast, the frequency of calls at the adolescent stage was significantly higher compared with infancy at P4 (Trial 1: p<0.001 for all tested strains; trial 2: C57BL/6N: p = 0.0096, DBA/2: p = 0.041 and FVB/N: p = 0.0092). Because of the difference of the arenas sizes and shapes, amplitudes of calls cannot be directly compared between adolescent and infant stages.

By analyzing the USV patterns, DBA/2 mice presented less heterogeneity in the USV categories than both FVB/N and C57BL/6N mice. DBA/2 mice emitted mainly downward calls during the habituation (trial 1) and social recognition (trial 2) (50%) and flat calls (40%) when the unfamiliar mouse was presented (trial 3) (Fig 3F). In all trials, the calls with a flat shape were the most frequent calls emitted by C57BL/6N mice (40%) (Fig 3F). For FVB/N mice, the USV patterns of trial 1 and trial 2 were more similar compared to trial 3 and represented by complex (> 35%), downwards (> 25%), flat (> 10%) and composites (> 10%) calls (Fig 3F). Interestingly, the harmonic calls (5%) were emitted only by FVB/N mice in trial 3. Moreover, the short calls were emitted only by C57BL/6N mice in trial 3.

### Ultrasonic vocalizations in adolescent mice in the direct social interaction test

#### Number of calls and calls characteristics

**Fig 4:**
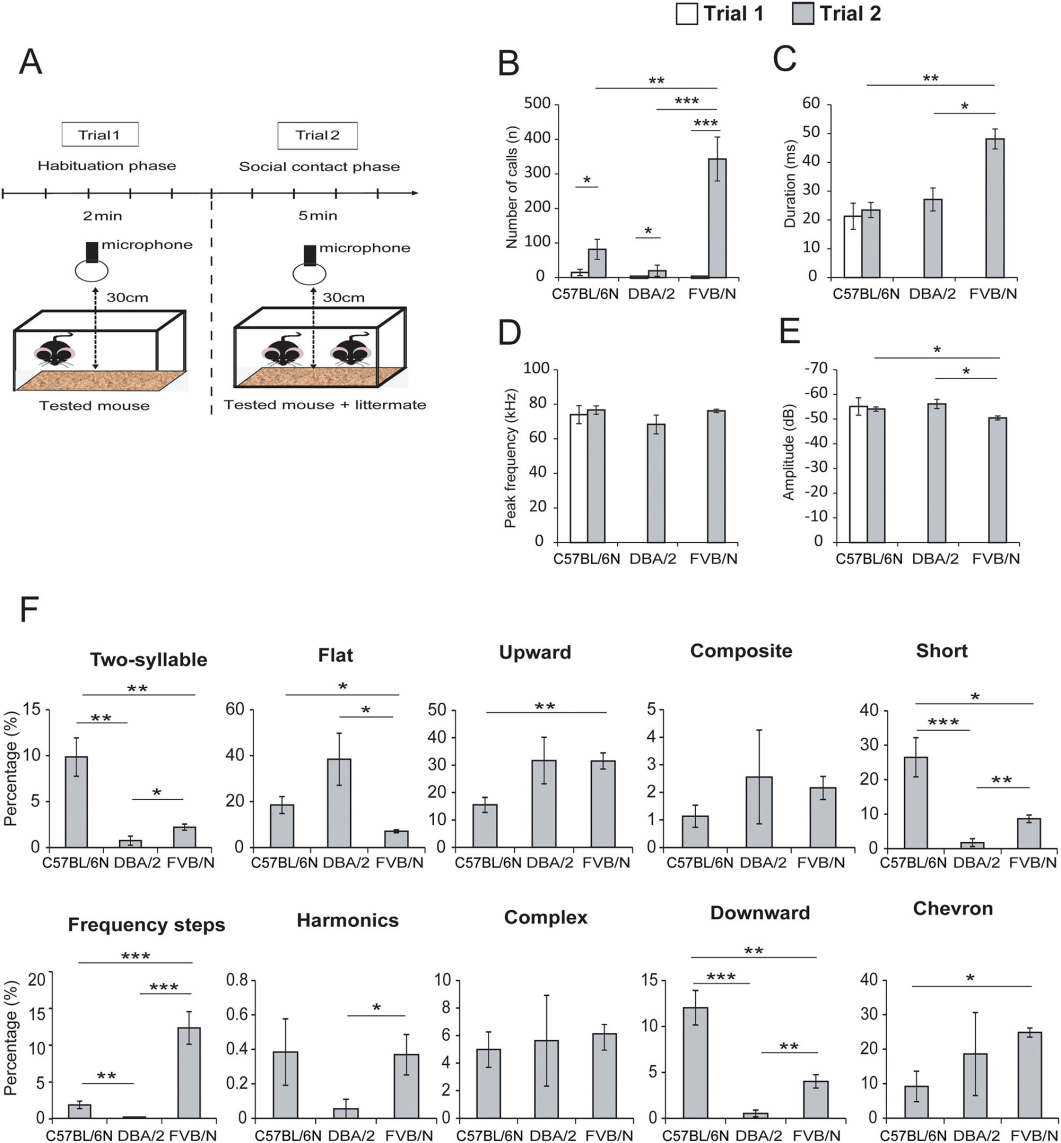
USV analysis of adolescent mice from C57BL/6N, DBA/2 and FVB/N strains during the direct social interaction test. **(A)** A schematic drawing of the experimental setup for USV recording in adolescent mice. USV were recorded for 2 min in a new arena as a habituation phase (trial 1), followed by a social contact phase for 5 min with a same-sex littermate (trial 2). **(B-E)** USV analysis including number **(B)**, duration **(C)**, frequency **(D)** and amplitude **(E)** of calls at P24 during the habituation and social contact phases. **(F)** Percentages of different calls categories emitted by each strain during social contact phase; n = 25 for C57BL/6N (14 males and 11 females), n = 14 for DBA/2 (7 males and 7 females) and n = 18 for FVB/N (7 males and 11 females), all data as mean ± SEM (two-way ANOVA, *p< 0.05, **p< 0.01, ***p< 0.001).

In this task, the USV profiles were measured during a direct social approach of 2 adolescent littermates of the same gender. An adolescent mouse was isolated from the litter in a home cage. After 24 h, the isolated mouse was put in a new arena for 2 min for habituation (habituation trial 1) followed by a social interaction for 5 min with a same-sex littermate (social interaction trial 2) (Fig 4A). In trial 1, C57BL/6N marginally emitted USV in contrast to DBA/2 and FVB/N (Fig 4B). In trial 2, adolescent mice from the three tested strains emitted USV when they were reunited with a littermate. Noticeably, the numbers of calls produced by the FVB/N mice were significantly more than the calls emitted by the C57BL/6N and DBA/2 mice (p = 0.003 vs C57BL/6N and 0.0007 vs DBA/2) (Fig 4B). In addition, the duration of calls was significantly longer in FVB/N compared with C57BL/6N mice (p = 0.0094 vs C57BL/6N and 0.012 vs DBA/2) (Fig 4C), but the peak frequencies were similar between all three strains (Fig 4D). FVB/N mice emitted louder calls than C57BL/6N and DBA/2 mice (Fig 4E). When considering the gender, no difference in the number, duration, frequency or amplitude of the USV calls was detected between male and female adolescent mice from the three tested strains (S4A Fig).

#### Call categories

The patterns of USV calls produced by the three tested strains during trial 2 of the direct social interaction test were investigated. The highest percentage of USV pattern emitted by C57BL/6N mice was short (≃ 30%) (Fig 4F). For DBA/2 and FVB/N adolescent mice, the highest percentages of calls patterns were flat (≃ 40%) and upward (≃ 30%), respectively (Fig 4F). The heterogeneity of the calls patterns in DBA/2 mice was less compared with the other two strains, similarly to the results obtained from the three-chamber social test. Moreover, DBA/2 mice did not emit any harmonic or frequency steps calls (Fig 4F). In addition, there was no gross difference in call categories between males and females mice except in DBA/2 mice (S4B Fig).

## Discussion

Because USV are extensively used nowadays in mouse models of neurological diseases including verbal dyspraxia, depression, autism and stuttering [47], their detailed analysis in wild-type mice compared to different strains at different developmental states is a must in order to better understand their basic characteristics under different conditions. This, in turn, can direct the choice of the best strain during a specific developmental state for the investigation of neuropsychiatric disorders. Although many studies have already shown that USV properties vary across strains [4, 30, 48, 49], social contexts [50–52] and between different ages especially pups and adults [53, 54], all of these parameters were not thoroughly compared in one single study. In the present comprehensive study, we analyzed many USV characteristics including the number, duration, frequency and amplitude in addition to the categories of calls in three wild-type mouse strains C57BL/6N, DBA/2 and FVB/N under different contexts in a longitudinal manner following the same mice from infancy to adolescence, in order to provide a more unified data set.

During infancy, when pups were separated from their dam and littermates, the USV properties varied across postnatal days and strains indicating an influence of developmental state and genetic background on the ultrasound emissions. When comparing the number of calls at P4, no difference was found between the strains. In contrast, differences in all USV characteristics can be observed at P8 and P12, suggesting that the differentiation in communication behavior might occur after P4. FVB/N pups vocalized longer USV, and the number of their calls decreased with age compared to C57BL/6N and DBA/2. Although the literature suggests a strain-specific peak in vocalization rate at around P8 [13, 48], this was not observed in the current study. The duration of calls decreased from P4 to P12 in all tested strains, which is consistent with previous studies that showed that the duration of calls decreases with ages [47, 54, 55]. Compared with other tested strains, The DBA/2 pups consistently displayed lower frequency and higher intensity calls.

The USV properties did not grossly differ between the contexts of pups separation and reunion with a littermate, which may indicate that the presence of a littermate is not sufficient to change the affective state of the separated pup. However, a current limitation of the spectrograms is the inability to distinguish which of the two pups vocalized more or which remained silent. Interestingly, studies in rats with anesthetized littermates introduced to the separated pups illustrated a reduction in vocalization rate [56]. Considering the fact that neonate mice are deaf till P10 [11], two assumptions can be made: (i) Both pups were unable to interact and hence both produced distress calls resulting in significantly increased number of USV or (ii) pups were able to establish contact (tactile or olfactory perception) leading to a reduction in ultrasound emissions. However, none of these hypotheses could be confirmed by the observed results leaving the open question whether contact between two conscious littermates changes the ultrasound emission.

The body weights and temperatures of pups can have an effect on the USV characteristics [57]. Therefore, these parameters were checked prior to experimentation and revealed reduced body weights of DBA/2 pups compared with C57BL/6N and FVB/N pups. However, the influence of smaller body weight on temperature control and ultrasound emission was excluded since all strains lost body temperature during separation at P4 and P8. This result was also confirmed by Scattoni et al. excluding the influence of body size on the vocalization rate [6].

Following these mice to the adolescent stage, the USV category profiles illustrated similarities across three different trials in the three-chamber social test. However, the first two trials displayed more similarities compared to the third one, especially with the number of calls in the three tested strains. Although we cannot exclude that some of the recorded calls were obtained from the littermate in trial 2 or the adult unfamiliar mouse in trial 3, we can reasonably consider that most of USV calls were emitted by the tested mouse since we did not observe overlapping vocalization streams, consistent with a single vocalizer and as the host mouse has been shown to be the main emitter of USV [58]. As the unfamiliar adult mice in trial 3 were from different strains: DBA/2 for the tested C57BL/6N strain; FVB/N for the tested DBA/2 strain and C57BL/6N for the tested FVB/N strain, the consistent differences in USV characteristics between trial 3 and both trial 1 and 2 in all tested strains may not be a consequence of individual variability and likely indicates an influence of the territorial interaction with an unfamiliar mouse versus a normal social interaction with a littermate. This confirms that mice are able to adapt their vocal behaviors in a context-dependent manner in order to convey different information [59]. The frequency of calls from all tested strains was very high during the habituation trial and decreased in the social recognition trial when there was a mouse in the arena, especially when it was an unfamiliar one from a different strain. The low-frequency calls during the exposure to an adult stranger mouse might be similar to the 22 kHz alarm calls emitted by rats in an aversive context [60, 61] [62]. Moreover, it has been shown that high-frequency calls were recorded during social interaction tasks, while the lowest frequency calls were recorded under stressful situations [3]. The duration of the USV calls in trial 2 and trial 3 (social trials, littermate or unfamiliar mouse, respectively) was similar to trial 1 (non-social trial, habituation) although it has been shown that the calls emitted in a social interaction task were longer in comparison with the calls emitted in other non-social situations [3].

A comparison of the USV characteristics between the adolescent and the infant stages revealed a decrease in the number of calls and their durations in all strains during adolescence, despite the longer duration of the experiment (5 min in the infant stage and 10 min in the adolescent stage). This suggests that the role of USV in survival as shown by calling the dam after separation is more vital than their role in social or territorial interactions. Additionally, adolescent mice from all tested strains emitted higher frequency calls in the habituation phase (trial 1) and social contact phase (trial 2) in the three-chamber social test than while they were infants after separation from the dam (trial 1) and reunion with a littermate (trial 2). This also confirms that the low-frequency calls indicate highly stressful situations which may present during infancy, in particular during the risk of hypothermia. However, apart from stress, other factors including the social context and normal development might explain the high-frequency calls during adolescence compared with the low-frequency calls during infancy.

To test the effects of the social context on the USV characteristics, we also recorded USV from another cohort of mice during the direct social interaction test. We did not perform this test on the same mice used during the three-chamber social test to avoid a previous social experience which can have a major effect on the pattern of the USV emission. In trial 1, when the mice were alone in an open arena, they hardly emitted any USV. In contrast, they emitted many USV calls in trial 2, when they were socially active with same-sex littermates, indicating a positive correlation between USV and the social approach. Compared with the three-chamber social test, the three tested strains emitted more ultrasound signals in the direct social test during the social recognition trial, although the test lasted shorter. The low vocalization rates observed in the three-chamber social test can be explained by the limited social interaction, given the presence of a barrier with the wire mesh cylinder and the bigger arena area, which would leave the tested mouse with less option to socialize directly. In contrast, a comparison of the duration and frequency of calls between the social contact phases (trial 2) in both the direct social interaction test and the three-chamber social test revealed no differences. In the direct social interaction test, the USV properties were different between different strains, with the FVB/N mice showing the highest number and duration of calls with reduced frequency and amplitude. Moreover, call categories were unique for each strain which confirms that the USV calls emitted by mice are a communication tool during different types of situations or contexts. Therefore, the USV patterns of adolescent mice can serve as indicators for strain-specific social approaches.

During both social interaction experiments, DBA/2 adolescent mice showed less USV calls, which can be partially explained by their hearing disability. This strain shows a high-frequency hearing loss at as early as 3 weeks of age caused by *Cdh23*^*753G>A*^ [63] and *Fscn2*^*326G>A*^ alleles [64] genetic variants. However, the significant role of optimal hearing ability on the emission of USV is still controversial. Adult mice from several strains have been shown to emit USV during courtship without an auditory feedback [65]. Moreover, previous studies have revealed that adult DBA/2 mice can emit USV to the same degree as other strains including CD1 and 129SV [58]. Therefore, other factors may have caused the reduced number of USV in juvenile DBA/2 mice. Therefore, C57BL/6N, DBA/2 and FVB/N should be further investigated in various social contexts during adulthood to check whether the reduced number of calls in DBA/2 mice is constant at different developmental stages.

In all experiments performed on the three tested strains during infancy and adolescence, we did not observe any significant differences between males and females in the number of calls or other USV characteristics consistent with studies performed on adult male and female mice [32] [66] [67] [68], indicating that the social communication between mice of the same sex could be considered as different from the reproductive context between opposite sex as shown previously [69].

Mice did not show clear visual cues of their vocal behavior during social interactions. Therefore, we were not able to confirm that the produced USV calls were mainly emitted by the tested mice during infancy or adolescence, which was the main limitation of our study. However, as the tested mice were the host mice that were habituated in the experimental devices before the introduction of a littermate or a stranger mouse, these mice were considered the main emitters of USV, as shown previously [58]. Additionally, in most of our spectrograms, we did not observe overlapping vocalization patterns, suggesting a single vocalizer. To avoid this dilemma in the future, the USV measurement should be accompanied by an accurate social behavior analysis using tracking software for better synchronization of the calls and confirmation of the emitter mouse. A recent study has proposed a new approach for a high-precision spatial localization of the mouse USV during social interaction test, which would allow a proper understanding of social vocalizations [66]. A detailed examination of the temporal and sequencing aspects of vocalizations bouts could further provide an insight into how particular patterns of vocalizations can confer different meanings.

The long-term study following the same individuals from the little to the juvenile age should reduce the variability within the study groups. Therefore, it could also be conceivable to measure USV every single day from P4 to the late adolescence at P40 to give more precise insights over the entire developmental progression. However, this may represent a high burden on mice, and the habituation effects due to the daily manipulation might additionally influence the results. To this end, we propose, in addition to the paradigm of pup isolation test and reunion, additional measurements of USV during adolescence which can be performed weekly until P40.

Here, we report for the first time a comprehensive analysis of USV by providing all calls characteristics from three different wild-type mouse strains in a longitudinal manner starting from infancy to adolescence. As observed for other behaviors with these three inbred mice, differences in USV characteristics can also be detected at different ages and social contexts using our paradigms. Together, this study gives a more complex picture of all variables that can modify USV. These variables need to be considered during the design and analysis of mouse models of mental disorders and/or communication deficits.

## Outlook

This study focused on a detailed USV analysis of different strains during isolation and reunion of pups and two social interaction contexts in adolescent mice. Other commonly used social interaction tests include social conditioned place preference, partition and operant condition tests [70]. The effects of these tests on the USV characteristics should be further investigated along with other kinds of behaviors in which mice emit USV like nursing, play and aggression. Moreover, the synchronization of different call characteristics with a specific type of social interactions is of great importance, in order to assign calls sequencing patterns with specific types of behaviors. This, in turn, will facilitate the examination of acoustic features that are related to specific biological states and social outcomes. In addition, it can determine the type of information encoded in the different call types and their functions [4, 29, 71, 72]. This can be carried out with the recent advancement of USV analysis from a manual to a total automated analysis using the new Mouse Ultrasonic Profile ExTraction (MUPET) technique [73]. With this technique, the USV repertoire can now be measured and compared, while providing an automated time-stamp of individual USV events. Such analysis tools will accelerate the progress of social communication studies in rodents [73].

## Supporting information

S1 Fig Body weight (A) and temperature (B) of pups during separation from the home cage at P4, P8 and P12. Body temperature was measured before separation (pre-experiment) and immediately after testing (post-experiment); n = 7 for C57BL/6N, n = 8 for DBA/2 and n = 11 for FVB/N, all data as mean ± SEM (two-way ANOVA, *p< 0.05, **p< 0.01).
(TIF)

S2 Fig Comparison of USV in infancy including the number, duration, frequency and amplitude of calls across P4, P8 and P12 during separation (trial 1) and reunion with a littermate (trial 2); n = 7 for C57BL/6N, n = 8 for DBA/2 and n = 11 for FVB/N, all data as mean ± SEM (two-way ANOVA, *p< 0.05, **p< 0.01, ***p< 0.001). (TIF)

S3 Fig Comparison of USV between male and female juvenile mice from the C57BL/6N, DBA/2 and FVB/N strains during the three-chamber social test. USV analysis includes the number, duration, frequency and amplitude of calls during three trials (habituation trial 1, social recognition trial 2 and social discrimination trial 3); n = 2 males and 5 females for C57BL/6N, n = 4 males and 4 females for DBA/2 and n = 6 males and 5 females for FVB/N, all data as mean ± SEM (two-way ANOVA).
(TIF)

S4 Fig Comparison of USV between male and female juvenile mice from the C57BL/6N, DBA/2 and FVB/N strains during the direct social interaction with a littermate after 24 h of isolation. **(A)** USV analysis including the number, duration, frequency and amplitude of calls at P24 during the social interaction phase. **(B)** Percentages of call categories emitted by males and females from each strain during the social interaction phase; n = 14 males and 11 females for C57BL/6N, n = 7 males and 7 females for DBA/2 and n = 7 males and 11 females for FVB/N, all data as mean ± SEM (two-way ANOVA, *p< 0.05, **p< 0.01, ***p< 0.001).
(TIF)

## References

[1] Wohr M, Schwarting RK. Affective communication in rodents: ultrasonic vocalizations as a tool for research on emotion and motivation. Cell Tissue Res. 2013;354(1):81–97. 10.1007/s00441-013-1607-9 . 23576070

[2] Mun HS, Lipina TV, Roder JC. Ultrasonic Vocalizations in Mice During Exploratory Behavior are Context-Dependent. Front Behav Neurosci. 2015;9:316 10.3389/fnbeh.2015.00316 26696847 PMC4674556

[3] Chabout J, Serreau P, Ey E, Bellier L, Aubin T, Bourgeron T, et al Adult male mice emit context-specific ultrasonic vocalizations that are modulated by prior isolation or group rearing environment. PLoS One. 2012;7(1):e29401 10.1371/journal.pone.0029401 22238608 PMC3253078

[4] Panksepp JB, Jochman KA, Kim JU, Koy JJ, Wilson ED, Chen Q, et al Affiliative behavior, ultrasonic communication and social reward are influenced by genetic variation in adolescent mice. PLoS One. 2007;2(4):e351 10.1371/journal.pone.0000351 17406675 PMC1831495

[5] Portfors CV. Types and functions of ultrasonic vocalizations in laboratory rats and mice. J Am Assoc Lab Anim Sci. 2007;46(1):28–34. . 17203913

[6] Scattoni ML, Gandhy SU, Ricceri L, Crawley JN. Unusual repertoire of vocalizations in the BTBR T+tf/J mouse model of autism. PLoS One. 2008;3(8):e3067 10.1371/journal.pone.0003067 18728777 PMC2516927

[7] Moles A, Costantini F, Garbugino L, Zanettini C, D’Amato FR. Ultrasonic vocalizations emitted during dyadic interactions in female mice: a possible index of sociability? Behav Brain Res. 2007;182(2):223–30. 10.1016/j.bbr.2007.01.020 . 17336405

[8] Egnor SR, Seagraves KM. The contribution of ultrasonic vocalizations to mouse courtship. Curr Opin Neurobiol. 2016;38:1–5. 10.1016/j.conb.2015.12.009 . 26789140

[9] Neunuebel JP, Taylor AL, Arthur BJ, Egnor SE. Female mice ultrasonically interact with males during courtship displays. Elife. 2015;4 10.7554/eLife.06203 26020291 PMC4447045

[10] Sewell GD. Ultrasonic communication in rodents. Nature. 1970;227(5256):410 10.1038/227410a0 . 5464098

[11] Ehret G. Infant rodent ultrasounds—a gate to the understanding of sound communication. Behav Genet. 2005;35(1):19–29. 10.1007/s10519-004-0853-8 . 15674530

[12] D’Amato FR, Scalera E, Sarli C, Moles A. Pups call, mothers rush: does maternal responsiveness affect the amount of ultrasonic vocalizations in mouse pups? Behav Genet. 2005;35(1):103–12. 10.1007/s10519-004-0860-9 . 15674537

[13] Scattoni ML, Crawley J, Ricceri L. Ultrasonic vocalizations: a tool for behavioural phenotyping of mouse models of neurodevelopmental disorders. Neurosci Biobehav Rev. 2009;33(4):508–15. 10.1016/j.neubiorev.2008.08.003 18771687 PMC2688771

[14] Lahvis GP, Alleva E, Scattoni ML. Translating mouse vocalizations: prosody and frequency modulation. Genes Brain Behav. 2011;10(1):4–16. 10.1111/j.1601-183X.2010.00603.x 20497235 PMC2936813

[15] Ey E, Torquet N, Le Sourd AM, Leblond CS, Boeckers TM, Faure P, et al The Autism ProSAP1/Shank2 mouse model displays quantitative and structural abnormalities in ultrasonic vocalisations. Behav Brain Res. 2013;256:677–89. 10.1016/j.bbr.2013.08.031 . 23994547

[16] Scattoni ML, McFarlane HG, Zhodzishsky V, Caldwell HK, Young WS, Ricceri L, et al Reduced ultrasonic vocalizations in vasopressin 1b knockout mice. Behav Brain Res. 2008;187(2):371–8. 10.1016/j.bbr.2007.09.034 18005969 PMC2255061

[17] Jamain S, Radyushkin K, Hammerschmidt K, Granon S, Boretius S, Varoqueaux F, et al Reduced social interaction and ultrasonic communication in a mouse model of monogenic heritable autism. Proc Natl Acad Sci U S A. 2008;105(5):1710–5. 10.1073/pnas.0711555105 18227507 PMC2234209

[18] Long JM, LaPorte P, Paylor R, Wynshaw-Boris A. Expanded characterization of the social interaction abnormalities in mice lacking Dvl1. Genes Brain Behav. 2004;3(1):51–62. . 14960015 10.1046/j.1601-183x.2003.00045.x

[19] Picker JD, Yang R, Ricceri L, Berger-Sweeney J. An altered neonatal behavioral phenotype in Mecp2 mutant mice. Neuroreport. 2006;17(5):541–4. 10.1097/01.wnr.0000208995.38695.2f . 16543822

[20] Wohr M, Silverman JL, Scattoni ML, Turner SM, Harris MJ, Saxena R, et al Developmental delays and reduced pup ultrasonic vocalizations but normal sociability in mice lacking the postsynaptic cell adhesion protein neuroligin2. Behav Brain Res. 2013;251:50–64. 10.1016/j.bbr.2012.07.024 22820233 PMC3979986

[21] Scattoni ML, Martire A, Cartocci G, Ferrante A, Ricceri L. Reduced social interaction, behavioural flexibility and BDNF signalling in the BTBR T+ tf/J strain, a mouse model of autism. Behav Brain Res. 2013;251:35–40. 10.1016/j.bbr.2012.12.028 . 23270976

[22] Schmeisser MJ, Ey E, Wegener S, Bockmann J, Stempel AV, Kuebler A, et al Autistic-like behaviours and hyperactivity in mice lacking ProSAP1/Shank2. Nature. 2012;486(7402):256–60. Epub 2012/06/16. 10.1038/nature11015 . 22699619

[23] Won H, Lee HR, Gee HY, Mah W, Kim JI, Lee J, et al Autistic-like social behaviour in Shank2-mutant mice improved by restoring NMDA receptor function. Nature. 2012;486(7402):261–5. Epub 2012/06/16. 10.1038/nature11208 . 22699620

[24] Radyushkin K, Hammerschmidt K, Boretius S, Varoqueaux F, El-Kordi A, Ronnenberg A, et al Neuroligin-3-deficient mice: model of a monogenic heritable form of autism with an olfactory deficit. Genes Brain Behav. 2009;8(4):416–25. 10.1111/j.1601-183X.2009.00487.x . 19243448

[25] Scearce-Levie K, Roberson ED, Gerstein H, Cholfin JA, Mandiyan VS, Shah NM, et al Abnormal social behaviors in mice lacking Fgf17. Genes Brain Behav. 2008;7(3):344–54. 10.1111/j.1601-183X.2007.00357.x . 17908176

[26] Dirks A, Fish EW, Kikusui T, van der Gugten J, Groenink L, Olivier B, et al Effects of corticotropin-releasing hormone on distress vocalizations and locomotion in maternally separated mouse pups. Pharmacol Biochem Behav. 2002;72(4):993–9. 10.1016/s0091-3057(02)00809-2 . 12062591

[27] Fish EW, Faccidomo S, Gupta S, Miczek KA. Anxiolytic-like effects of escitalopram, citalopram, and R-citalopram in maternally separated mouse pups. J Pharmacol Exp Ther. 2004;308(2):474–80. 10.1124/jpet.103.058206 . 14593091

[28] Fish EW, Sekinda M, Ferrari PF, Dirks A, Miczek KA. Distress vocalizations in maternally separated mouse pups: modulation via 5-HT(1A), 5-HT(1B) and GABA(A) receptors. Psychopharmacology (Berl). 2000;149(3):277–85. . 10823409 10.1007/s002130000370

[29] Branchi I, Santucci D, Vitale A, Alleva E. Ultrasonic vocalizations by infant laboratory mice: a preliminary spectrographic characterization under different conditions. Dev Psychobiol. 1998;33(3):249–56. . 9810475 10.1002/(sici)1098-2302(199811)33:3<249::aid-dev5>3.0.co;2-r

[30] Sugimoto H, Okabe S, Kato M, Koshida N, Shiroishi T, Mogi K, et al A role for strain differences in waveforms of ultrasonic vocalizations during male-female interaction. PLoS One. 2011;6(7):e22093 10.1371/journal.pone.0022093 21818297 PMC3144874

[31] Burke K, Screven LA, Dent ML. CBA/CaJ mouse ultrasonic vocalizations depend on prior social experience. PLoS One. 2018;13(6):e0197774 10.1371/journal.pone.0197774 29874248 PMC5991354

[32] Heckman J, McGuinness B, Celikel T, Englitz B. Determinants of the mouse ultrasonic vocal structure and repertoire. Neurosci Biobehav Rev. 2016;65:313–25. 10.1016/j.neubiorev.2016.03.029 . 27060755

[33] Kikusui T, Nakanishi K, Nakagawa R, Nagasawa M, Mogi K, Okanoya K. Cross fostering experiments suggest that mice songs are innate. PLoS One. 2011;6(3):e17721 10.1371/journal.pone.0017721 21408017 PMC3052373

[34] Yang M, Loureiro D, Kalikhman D, Crawley JN. Male mice emit distinct ultrasonic vocalizations when the female leaves the social interaction arena. Front Behav Neurosci. 2013;7:159 10.3389/fnbeh.2013.00159 24312027 PMC3832782

[35] von Merten S, Hoier S, Pfeifle C, Tautz D. A role for ultrasonic vocalisation in social communication and divergence of natural populations of the house mouse (Mus musculus domesticus). PLoS One. 2014;9(5):e97244 10.1371/journal.pone.0097244 24816836 PMC4016290

[36] Dou X, Shirahata S, Sugimoto H. Functional clustering of mouse ultrasonic vocalization data. PLoS One. 2018;13(5):e0196834 10.1371/journal.pone.0196834 29742174 PMC5942836

[37] Moy SS, Nadler JJ, Young NB, Perez A, Holloway LP, Barbaro RP, et al Mouse behavioral tasks relevant to autism: phenotypes of 10 inbred strains. Behav Brain Res. 2007;176(1):4–20. 10.1016/j.bbr.2006.07.030 16971002 PMC1857288

[38] Logue SF, Paylor R, Wehner JM. Hippocampal lesions cause learning deficits in inbred mice in the Morris water maze and conditioned-fear task. Behav Neurosci. 1997;111(1):104–13. . 9109628 10.1037//0735-7044.111.1.104

[39] Logue SF, Owen EH, Rasmussen DL, Wehner JM. Assessment of locomotor activity, acoustic and tactile startle, and prepulse inhibition of startle in inbred mouse strains and F1 hybrids: implications of genetic background for single gene and quantitative trait loci analyses. Neuroscience. 1997;80(4):1075–86. 10.1016/s0306-4522(97)00164-4 . 9284061

[40] Owen EH, Logue SF, Rasmussen DL, Wehner JM. Assessment of learning by the Morris water task and fear conditioning in inbred mouse strains and F1 hybrids: implications of genetic background for single gene mutations and quantitative trait loci analyses. Neuroscience. 1997;80(4):1087–99. 10.1016/s0306-4522(97)00165-6 . 9284062

[41] Brooks SP, Pask T, Jones L, Dunnett SB. Behavioural profiles of inbred mouse strains used as transgenic backgrounds. II: cognitive tests. Genes Brain Behav. 2005;4(5):307–17. 10.1111/j.1601-183X.2004.00109.x . 16011577

[42] Brooks SP, Pask T, Jones L, Dunnett SB. Behavioural profiles of inbred mouse strains used as transgenic backgrounds. I: motor tests. Genes Brain Behav. 2004;3(4):206–15. 10.1111/j.1601-183X.2004.00072.x . 15248866

[43] Andre JM, Cordero KA, Gould TJ. Comparison of the performance of DBA/2 and C57BL/6 mice in transitive inference and foreground and background contextual fear conditioning. Behav Neurosci. 2012;126(2):249–57. 10.1037/a0027048 22309443 PMC3314134

[44] Taketo M, Schroeder AC, Mobraaten LE, Gunning KB, Hanten G, Fox RR, et al FVB/N: an inbred mouse strain preferable for transgenic analyses. Proc Natl Acad Sci U S A. 1991;88(6):2065–9. 10.1073/pnas.88.6.2065 1848692 PMC51169

[45] Pugh PL, Ahmed SF, Smith MI, Upton N, Hunter AJ. A behavioural characterisation of the FVB/N mouse strain. Behav Brain Res. 2004;155(2):283–9. 10.1016/j.bbr.2004.04.021 . 15364488

[46] Moy SS, Nadler JJ, Perez A, Barbaro RP, Johns JM, Magnuson TR, et al Sociability and preference for social novelty in five inbred strains: an approach to assess autistic-like behavior in mice. Genes, brain, and behavior. 2004;3(5):287–302. Epub 2004/09/04. 10.1111/j.1601-1848.2004.00076.x . 15344922

[47] Barnes TD, Rieger MA, Dougherty JD, Holy TE. Group and Individual Variability in Mouse Pup Isolation Calls Recorded on the Same Day Show Stability. Front Behav Neurosci. 2017;11:243 10.3389/fnbeh.2017.00243 29326565 PMC5736564

[48] Thornton LM, Hahn ME, Schanz N. Genetic and developmental influences on infant mouse ultrasonic calling. III. Patterns of inheritance in the calls of mice 3–9 days of age. Behav Genet. 2005;35(1):73–83. 10.1007/s10519-004-0857-4 . 15674534

[49] Scattoni ML, Ricceri L, Crawley JN. Unusual repertoire of vocalizations in adult BTBR T+tf/J mice during three types of social encounters. Genes Brain Behav. 2011;10(1):44–56. 10.1111/j.1601-183X.2010.00623.x 20618443 PMC2972364

[50] Chabout J, Sarkar A, Dunson DB, Jarvis ED. Male mice song syntax depends on social contexts and influences female preferences. Front Behav Neurosci. 2015;9:76 10.3389/fnbeh.2015.00076 25883559 PMC4383150

[51] Chabout J, Sarkar A, Patel SR, Radden T, Dunson DB, Fisher SE, et al A Foxp2 Mutation Implicated in Human Speech Deficits Alters Sequencing of Ultrasonic Vocalizations in Adult Male Mice. Front Behav Neurosci. 2016;10:197 10.3389/fnbeh.2016.00197 27812326 PMC5071336

[52] Hanson JL, Hurley LM. Female presence and estrous state influence mouse ultrasonic courtship vocalizations. PLoS One. 2012;7(7):e40782 10.1371/journal.pone.0040782 22815817 PMC3399843

[53] Grimsley JM, Monaghan JJ, Wenstrup JJ. Development of social vocalizations in mice. PLoS One. 2011;6(3):e17460 10.1371/journal.pone.0017460 21408007 PMC3052362

[54] Liu RC, Miller KD, Merzenich MM, Schreiner CE. Acoustic variability and distinguishability among mouse ultrasound vocalizations. J Acoust Soc Am. 2003;114(6 Pt 1):3412–22. 10.1121/1.1623787 . 14714820

[55] Hahn ME, Karkowski L, Weinreb L, Henry A, Schanz N, Hahn EM. Genetic and developmental influences on infant mouse ultrasonic calling. II. Developmental patterns in the calls of mice 2–12 days of age. Behav Genet. 1998;28(4):315–25. . 9803024 10.1023/a:1021679615792

[56] Hofer MA, Shair H. Ultrasonic vocalization during social interaction and isolation in 2-weeek-old rats. Dev Psychobiol. 1978;11(5):495–504. 10.1002/dev.420110513 . 689298

[57] Hahn ME, Lavooy MJ. A review of the methods of studies on infant ultrasound production and maternal retrieval in small rodents. Behav Genet. 2005;35(1):31–52. 10.1007/s10519-004-0854-7 . 15674531

[58] Faure A, Pittaras E, Nosjean A, Chabout J, Cressant A, Granon S. Social behaviors and acoustic vocalizations in different strains of mice. Behav Brain Res. 2017;320:383–90. Epub 2016/11/09. 10.1016/j.bbr.2016.11.003 . 27825934

[59] Portfors CV, Perkel DJ. The role of ultrasonic vocalizations in mouse communication. Curr Opin Neurobiol. 2014;28:115–20. 10.1016/j.conb.2014.07.002 25062471 PMC4177333

[60] Brudzynski SM. Ultrasonic calls of rats as indicator variables of negative or positive states: acetylcholine-dopamine interaction and acoustic coding. Behav Brain Res. 2007;182(2):261–73. 10.1016/j.bbr.2007.03.004 . 17467067

[61] Borta A, Wohr M, Schwarting RK. Rat ultrasonic vocalization in aversively motivated situations and the role of individual differences in anxiety-related behavior. Behav Brain Res. 2006;166(2):271–80. 10.1016/j.bbr.2005.08.009 . 16213033

[62] Schwarting RK, Wohr M. On the relationships between ultrasonic calling and anxiety-related behavior in rats. Braz J Med Biol Res. 2012;45(4):337–48. 10.1590/S0100-879X2012007500038 22437483 PMC3854164

[63] Noben-Trauth K, Zheng QY, Johnson KR. Association of cadherin 23 with polygenic inheritance and genetic modification of sensorineural hearing loss. Nat Genet. 2003;35(1):21–3. 10.1038/ng1226 12910270 PMC2864026

[64] Shin JB, Longo-Guess CM, Gagnon LH, Saylor KW, Dumont RA, Spinelli KJ, et al The R109H variant of fascin-2, a developmentally regulated actin crosslinker in hair-cell stereocilia, underlies early-onset hearing loss of DBA/2J mice. J Neurosci. 2010;30(29):9683–94. 10.1523/JNEUROSCI.1541-10.2010 20660251 PMC2922854

[65] Mahrt EJ, Perkel DJ, Tong L, Rubel EW, Portfors CV. Engineered deafness reveals that mouse courtship vocalizations do not require auditory experience. J Neurosci. 2013;33(13):5573–83. 10.1523/JNEUROSCI.5054-12.2013 . 23536072 PMC3691057

[66] Heckman JJ, Proville R, Heckman GJ, Azarfar A, Celikel T, Englitz B. High-precision spatial localization of mouse vocalizations during social interaction. Sci Rep. 2017;7(1):3017 10.1038/s41598-017-02954-z 28592832 PMC5462771

[67] Hammerschmidt K, Radyushkin K, Ehrenreich H, Fischer J. The structure and usage of female and male mouse ultrasonic vocalizations reveal only minor differences. PLoS One. 2012;7(7):e41133 10.1371/journal.pone.0041133 22815941 PMC3398926

[68] Briggs JR, Kalcounis-Rueppell MC. Similar acoustic structure and behavioural context of vocalizations produced by male and female California mice in the wild. Animal Behaviour. 2011;82(6):1263–73. 10.1016/j.anbehav.2011.09.003.

[69] Zala SM, Reitschmidt D, Noll A, Balazs P, Penn DJ. Sex-dependent modulation of ultrasonic vocalizations in house mice (Mus musculus musculus). PloS one. 2017;12(12):e0188647–e. 10.1371/journal.pone.0188647 . 29236704 PMC5728457

[70] Eltokhi A, Rappold G, Sprengel R. Distinct Phenotypes of Shank2 Mouse Models Reflect Neuropsychiatric Spectrum Disorders of Human Patients With SHANK2 Variants. Frontiers in Molecular Neuroscience. 2018;11(240). 10.3389/fnmol.2018.00240 30072871 PMC6060255

[71] Wang H, Liang S, Burgdorf J, Wess J, Yeomans J. Ultrasonic vocalizations induced by sex and amphetamine in M2, M4, M5 muscarinic and D2 dopamine receptor knockout mice. PLoS One. 2008;3(4):e1893 10.1371/journal.pone.0001893 18382674 PMC2268741

[72] Holy TE, Guo Z. Ultrasonic songs of male mice. PLoS Biol. 2005;3(12):e386 10.1371/journal.pbio.0030386 16248680 PMC1275525

[73] Van Segbroeck M, Knoll AT, Levitt P, Narayanan S. MUPET-Mouse Ultrasonic Profile ExTraction: A Signal Processing Tool for Rapid and Unsupervised Analysis of Ultrasonic Vocalizations. Neuron. 2017;94(3):465–85 e5. 10.1016/j.neuron.2017.04.005 28472651 PMC5939957

