# Correction: Longitudinal analysis of ultrasonic vocalizations in mice from infancy to adolescence: Insights into the vocal repertoire of three wild-type strains in two different social contexts

**DOI:** 10.1371/journal.pone.0221469

**Published:** 2019-08-15

**Authors:** The PLOS ONE Staff

Corresponding author Ahmed Eltokhi’s email address is not included in the published article. The publisher apologizes for this error. Dr. Eltokhi’s email address is: ahmed.eltokhi.1@gmail.com.
