# Supplementary figures and images for: Longitudinal analysis of ultrasonic vocalizations in mice from infancy to adolescence: Insights into the vocal repertoire of three wild-type strains in two different social contexts
Source: PLoS One. 2019 Jul 31;14(7):e0220238. doi: 10.1371/journal.pone.0220238 (PMC6668806; doi:10.1371/journal.pone.0220238)

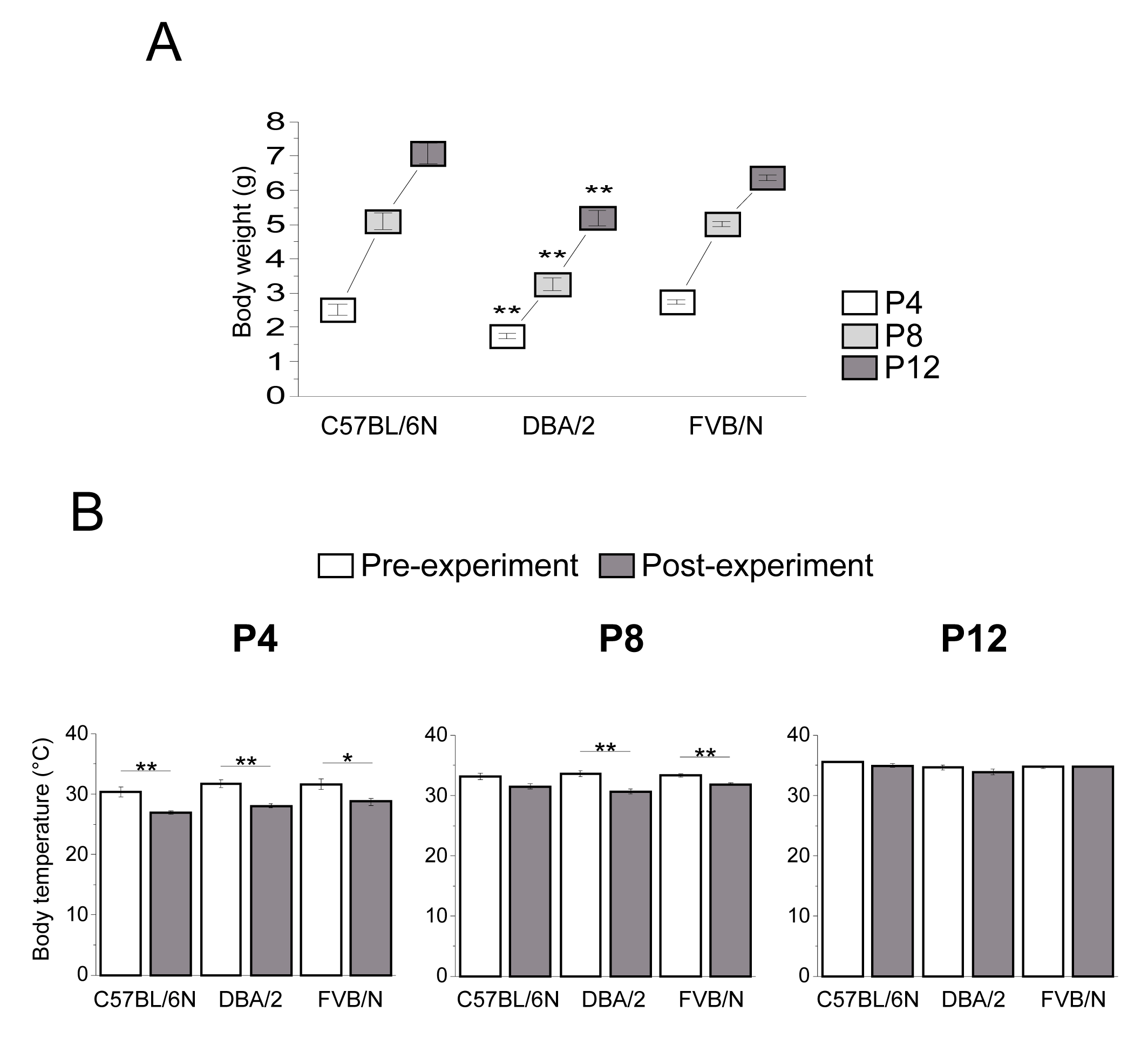

Supplement: S1 Fig — Body temperature was measured before separation (pre-experiment) and immediately after testing (post-experiment); n = 7 for C57BL/6N, n = 8 for DBA/2 and n = 11 for FVB/N, all data as mean ± SEM (two-way ANOVA, *p< 0.05, **p< 0.01). (TIF) [file pone.0220238.s001.tif]

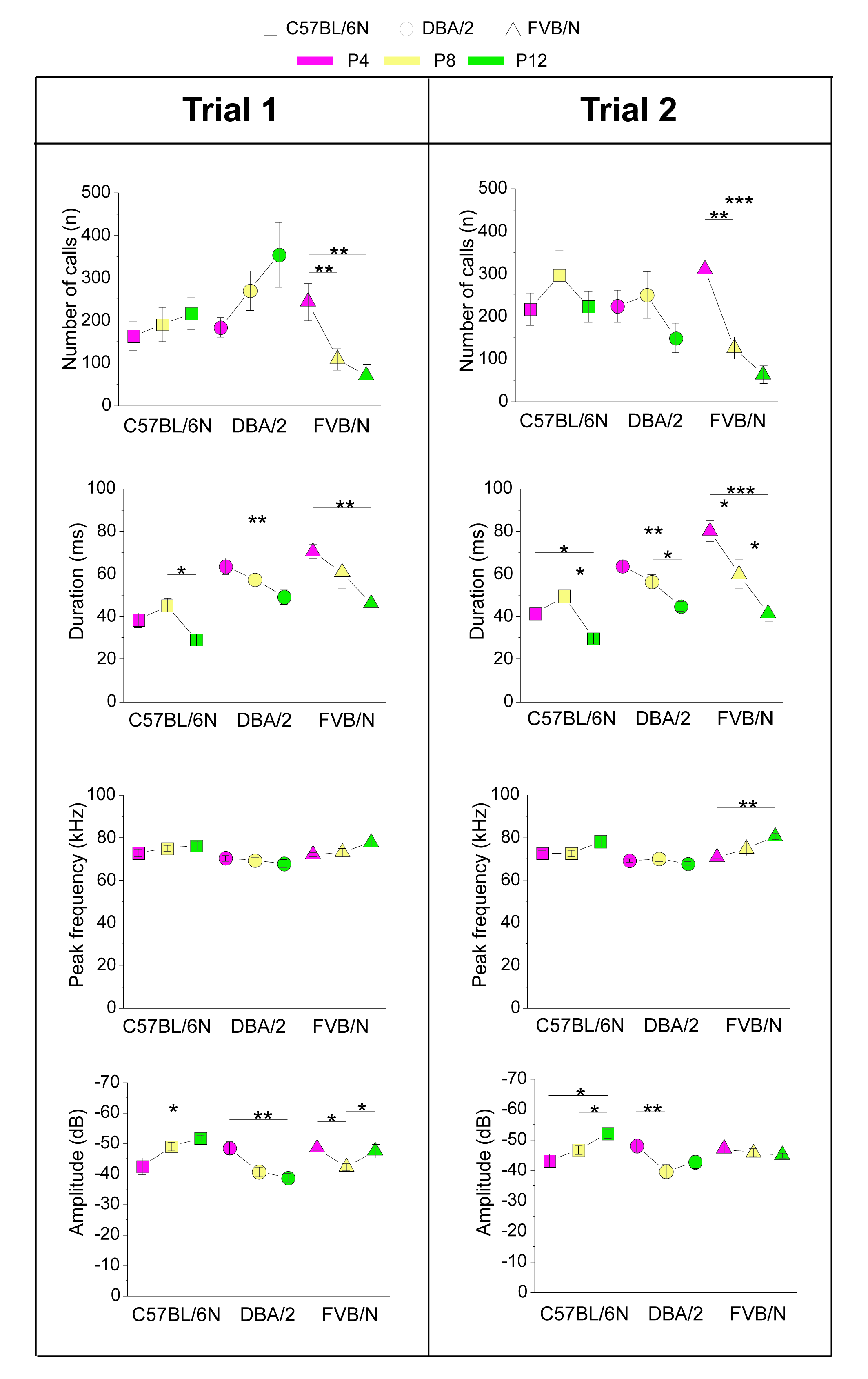

Supplement: S2 Fig — (TIF) [file pone.0220238.s002.tif]

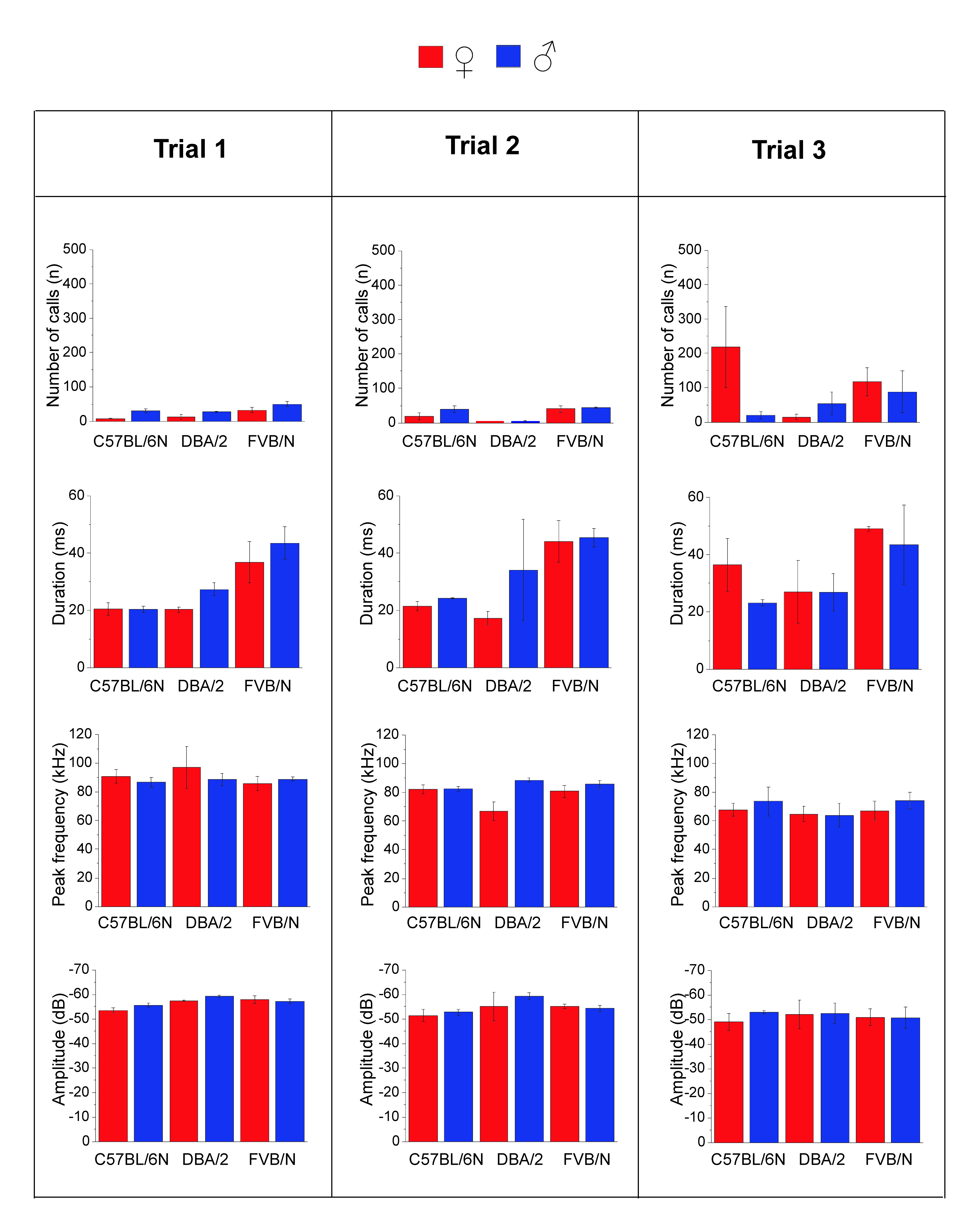

Supplement: S3 Fig — USV analysis includes the number, duration, frequency and amplitude of calls during three trials (habituation trial 1, social recognition trial 2 and social discrimination trial 3); n = 2 males and 5 females for C57BL/6N, n = 4 males and 4 females for DBA/2 and n = 6 males and 5 females for FVB/N, all data as mean ± SEM (two-way ANOVA). (TIF) [file pone.0220238.s003.tif]

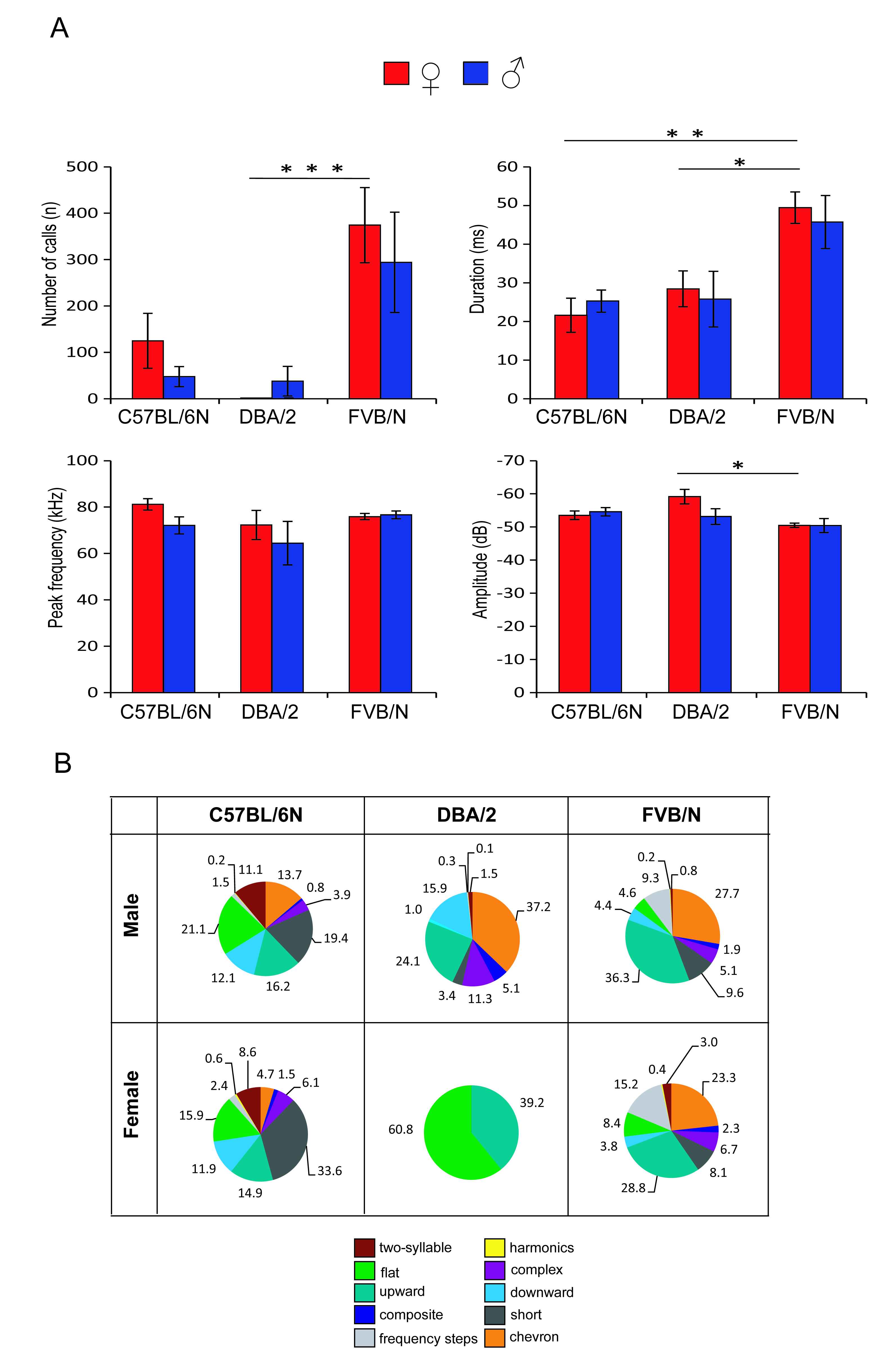

Supplement: S4 Fig — (A) USV analysis including the number, duration, frequency and amplitude of calls at P24 during the social interaction phase. (B) Percentages of call categories emitted by males and females from each strain during the social interaction phase; n = 14 males and 11 females for C57BL/6N, n = 7 males and 7 females for DBA/2 and n = 7 males and 11 females for FVB/N, all data as mean ± SEM (two-way ANOVA, *p< 0.05, **p< 0.01, ***p< 0.001). (TIF) [file pone.0220238.s004.tif]
